# Compositional Analysis of Ternary and Binary Chemical Mixtures by Surface-Enhanced Raman Scattering at Trace Levels

**DOI:** 10.1186/s11671-015-1142-6

**Published:** 2015-11-09

**Authors:** Mengjing Hou, Yu Huang, Lingwei Ma, Zhengjun Zhang

**Affiliations:** State Key Laboratory of New Ceramics and Fine Processing, School of Materials Science and Engineering, Tsinghua University, Beijing, 100084 People’s Republic of China; Key Laboratory of Advanced Materials (MOE), School of Materials Science and Engineering, Tsinghua University, Beijing, 100084 People’s Republic of China

**Keywords:** Principal component analysis, Compositional analysis, Surface-enhanced Raman scattering, Triangle-rule, Balance-rule

## Abstract

Surface-enhanced Raman scattering has been proven a powerful means in the fast detection and recognition of chemicals at trace levels, while quantitative analysis especially the compositional analysis of trace chemical mixtures remains a challenge. We report here a “triangle-rule” based on the principal component analysis (PCA) of surface-enhanced Raman scattering spectra, to calculate the composition of individual component of ternary chemical mixtures at trace levels, which can be simplified into the “balance-rule” for binary mixtures. We demonstrated the validity of the triangle-rule and balance-rule in estimating the composition of ternary and binary mixtures of methyl orange, methylene blue, and crystal violet with different molecular structures, and the validity for ternary and binary mixtures of three isomers of monochlorobiphenyl with very similar molecular structures. This idea might be also applicable to mixtures of more components at the trace levels.

## Background

Trace chemical detection is of great importance in the fields of environmental science, food science and medicine, etc. Recently, surface-enhanced Raman scattering (SERS) has been considered a powerful means as it is simple, fast, and is capable of recognizing molecules according to their vibrational fingerprints which are unique for a specific molecule [1–6]. It has been found that when a molecule is adsorbed on the surface of nanostructures of noble metals like Au and Ag, its Raman cross section can be greatly enhanced due to the localized surface plasmon resonance (LSPR) and the charge transfer between the molecule and nanostructure, namely the electromagnetic and chemical enhancements [7–11]. This leads to a great magnification of Raman signals of the molecule and makes it detectable at trace levels by Raman scattering [12, 13]. Therefore, SERS has become a promising qualitative tool in detecting and recognizing molecules and chemicals down to trace levels [14, 15].

In recent years, chemometrics methods, e.g., the principal component analysis (PCA), the hierarchical cluster analysis (HCA), partial least squares discriminant analysis (PLS-DA), etc., have been employed to identify components of the analyte with SERS spectra [16–19]. For example, Zhang et al. confirmed the existence of furazolidone and malachite green in fish products by the PCA of the SERS spectra [20]. Lin et al. discriminated the blood serum of colorectal cancer patients from that of healthy subjects by principal component analysis-linear discriminant analysis (PCA-LDA) of the SERS spectra [21]. Rivera-Betancourt et al. compared the validity of PCA, HCA, and PLS-DA in identifying mycobacteria [22]. In addition, chemometrics methods such as partial least squares regression (PLSR) and principal components regression (PCR) have been employed to correlate the amount of a molecule to its SERS spectra [23]. For instance, Zhai et al. measured ractopamine in swine urine with PLSR, and 0.4 μg/mL ractopamine could be detected [24]. Manikas et al. measured the SERS spectra of mitoxantrone solutions and found that their concentration can be well predicted in a range of 0 to 13 ng/mL by PLSR of the SERS spectra [25]. Both methods, however, are complicated and time-consuming, as the SERS spectra of a great amount of standard samples of various concentrations should be measured for the modeling process. New methodologies are therefore highly demanded for the quantitative analysis especially for the compositional analysis of chemical mixtures at trace levels by SERS.

In this study, we reported a simple method to calculate the compositions of ternary and binary chemical mixtures at trace levels by the PCA of the SERS spectra, which has been used previously to identify qualitatively the components in chemical mixtures. Based on the PCA analysis of the SERS spectra, we proposed a “triangle-rule” and a “balance-rule” for the ternary and binary mixtures, respectively, and demonstrated the validity of two rules in calculating the composition of two ternary model systems.

## Methods

### Fabrication of SERS Substrate

SERS substrate used in this research is silica nanorod (NR) array decorated with gold nanoparticles (NPs). SiO_2_ NRs were deposited on wafer with a DZS-500 electronic beam evaporation system (SKY Technology Development Co., Ltd. Chinese Academy of Sciences). To fabricate NRs perpendicular to wafer, glancing angle deposition (GLAD) technique was adopted. The incident angle of SiO_2_ beam was 86°, and the wafer kept in-plane rotation in the speed of 2 rpm. The SiO_2_ NRs were about 150 nm in height, with a diameter of around 30 nm. Au NPs were then sputtered on the NR array by a SBC-12 vacuum ion coater (KYKY Technology Co., Ltd.). The sputtering current was 10 mA, and the depositing time was 120 s. Consequently, the top and sidewall parts of SiO_2_ NRs were covered with great amount of Au NPs. The morphology of SiO_2_ NRs@Au NPs SERS substrate was characterized by scanning electron microscope (SEM, JEOL-JMS-7001F) and high-resolution transmission electron microscope (HRTEM, JEOL-2011).

### Preparation of Analytes

Methyl orange (MO), methylene blue (MB), and crystal violet (CV) are common dyes which were employed as SERS probe molecules. Each kind of dye powder was dissolved in deionized water and diluted to 5 × 10^−6^ M. Eight kinds of dye mixture solution listed in Table 1 were also prepared, and the total dye concentration of the mixtures was all 5 × 10^−6^ M. 2-Chlorobiphenyl (2-CB), 3-chlorobiphenyl (3-CB), and 4-chlorobiphenyl (4-CB) are isomers, which are similar in molecular structure. Their solution in acetone of the concentration of 5 × 10^−5^ M was prepared, respectively. The mixture of 2-CB and 3-CB with the ratio of 1:1 and the mixture of the three isomers with the ratio of 1:1:1 were prepared, and the total CB concentration of the mixture solution was both 5 × 10^−5^ M.

**Table 1:** Real compositions of the dye mixture samples

Sample	MO composition (%)	MB composition (%)	CV composition (%)	*X*_MO_:*X*_MB_:*X*_CV_
A1	50	50	0	1:1:0
A2	50	0	50	1:0:1
A3	0	50	50	0:1:1
A4	75	25	0	3:1:0
A5	0	75	25	0:3:1
A6	33.33	33.33	33.33	1:1:1
A7	66.66	16.67	16.67	4:1:1
A8	16.67	66.67	16.67	1:4:1

### Measurements of the SERS Spectra

SERS spectra of all the probe molecules above were measured with an optical fiber micro-Raman system (i-Raman Plus, B&W TEK Inc.), using a 785-nm laser as the excitation source. The laser power was 300 mW, and the beam spot was about 85 μm in diameter. Before Raman spectra of dyes were acquired, the SERS substrate was immersed in the solution for 30 min to make the dye molecules adsorbed, and dried naturally in air. The integral time of the dyes’ spectra was 5 s. CB molecules were adsorbed on the “hot spots” through the process of dropping 3 μL solution on every piece of SERS substrate. SERS spectra were obtained after the acetone volatilized completely, and the integral time was 20 s. SERS spectra were acquired at ten different areas randomly selected on each sample so that more accurate chemometrics model could be established basing on these data.

## Results and Discussion

**Fig. 1:**
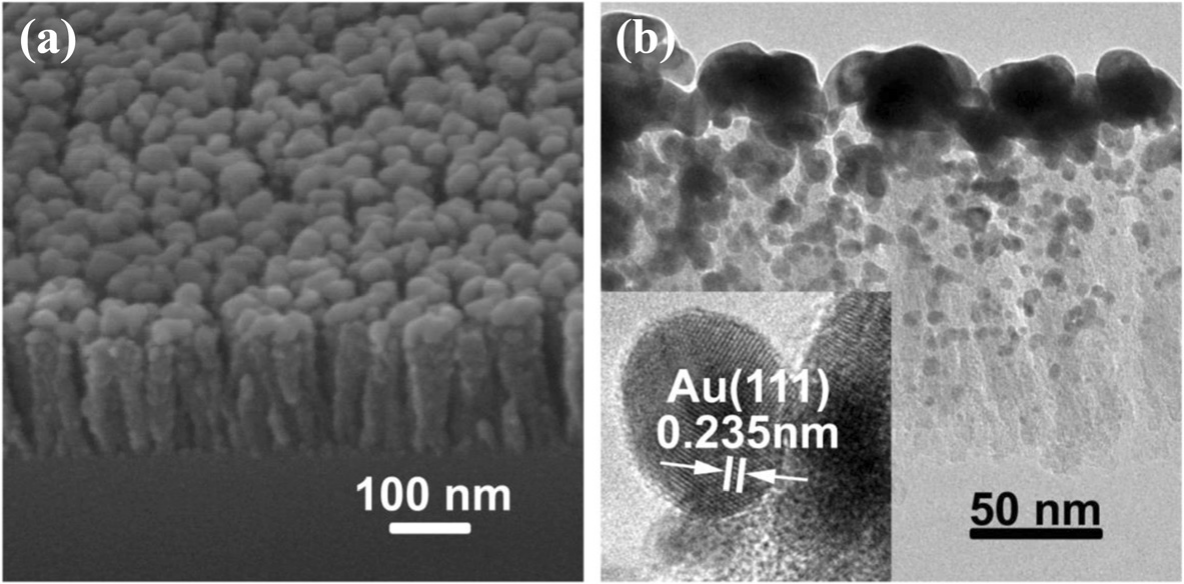
**a** SEM morphology of the SiO_2_ NRs@Au NPs SERS substrate. **b** TEM morphology of the SiO_2_ NRs@Au NPs SERS substrate. The *inset* is the HRTEM of Au NP decorated on SiO_2_ NR

Figure 1a, b shows, respectively, the SEM image and the TEM image of the SiO_2_ NRs@Au NPs SERS substrate. The inset of Fig. 1b is a typical HRTEM image of the Au NPs. One sees that the SiO_2_ NRs@Au NPs are well separated, the SiO_2_ nanorods are of a diameter of several 10 nm and a length of about 150 nm, and the Au NPs are of a diameter of typically several nanometers. These features suggest that this substrate should be of excellent SERS performance [26].

**Fig. 2:**
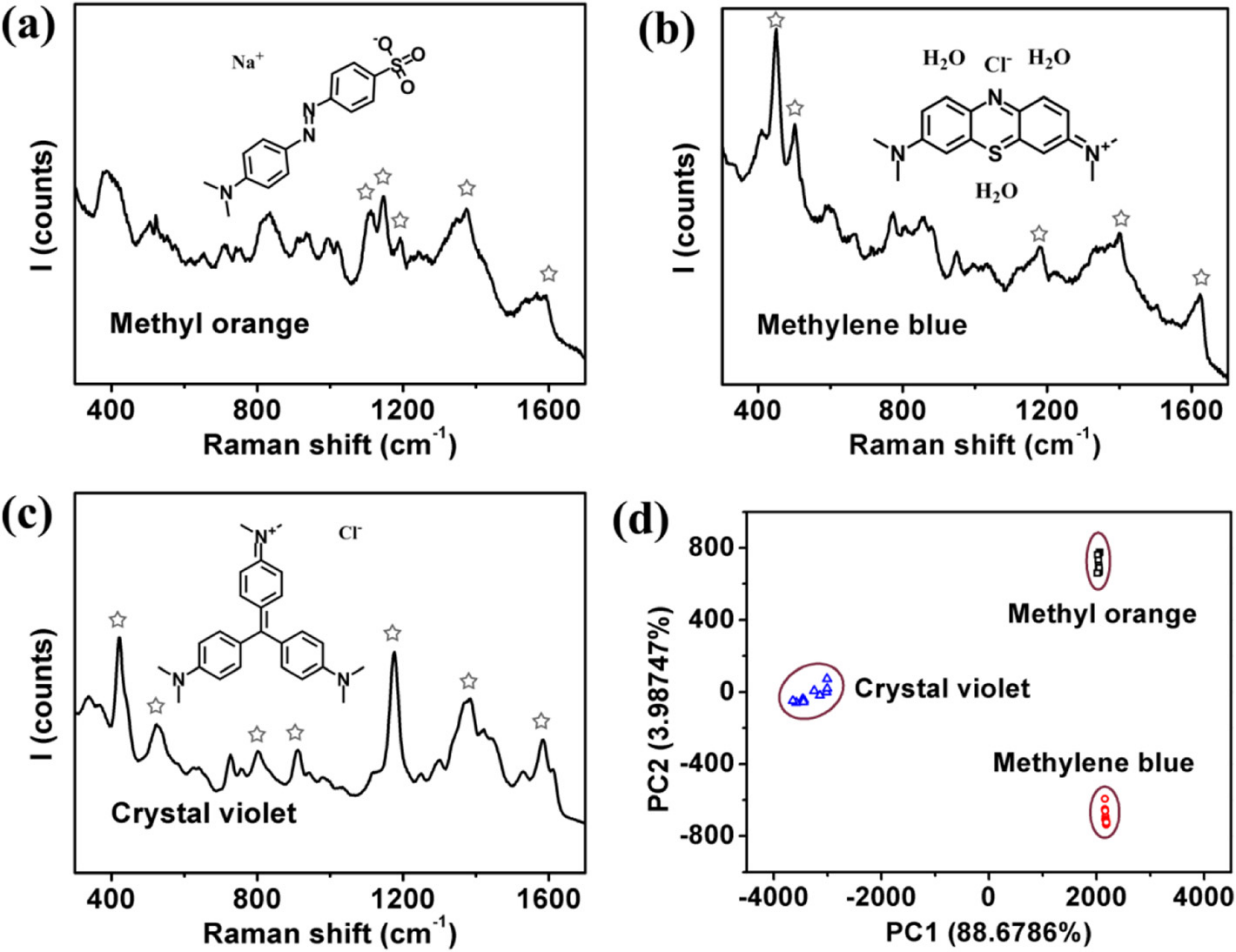
Typical SERS spectrum of 5 × 10^−6^ M **a** methyl orange, **b** methylene blue, and **c** crystal violet. **d** PCA score plot modeled by the SERS spectra of 5 × 10^−6^ M MO, MB, and CV

Figure 2a–c shows, respectively, the SERS spectra of 5 × 10^−6^ M solutions of MO, MB, and CV in water. The insets are structure models of three molecules. Figure 2a shows the characteristic peaks of MO at Raman shifts of ~1111, 1146, 1192, 1374, and 1590 cm^−1^, corresponding to the C-C bending, C-S stretching, N-N in-plane bending, C-C stretching, and C-C in-plane bending modes, respectively [27–29]. Figure 2b shows the characteristic peaks of MB at Raman shifts of ~449 and ~502 cm^−1^ of the C-N-C skeletal bending mode, ~1181 cm^−1^ of the C-N stretching mode, ~1400 cm^−1^ due to the deformation of CH3, and ~1624 cm^−1^ due to the stretching vibration of the ring, respectively [30–32]. Figure 2c shows the characteristic peaks of CV at Raman shifts of ~420, 523, 803, 910, 1174, 1383, and 1581 cm^−1^, corresponding to the phenyl-C-phenyl out-of-plane bending, C-N-C bending, phenyl-H out-of-plane bending, phenyl ring breathing, C-H in-plane ring deformation, C-N stretching, and phenyl ring stretching vibration, respectively [33–36]. As shown by the models, the three molecules are of different molecule structure, thus exhibited different SERS spectra. For a simpler and clearer comparison, PCA of the SERS spectra of the three molecules was carried out and the results are shown in Fig. 2d. Ten SERS spectra were used for PCA of each molecule, obtained from different places of the substrate. It is noted that by PCA, the three molecules can be clearly recognized by the scores of the first two principal components, i.e., PC1 and PC2. Another feature is that the score data of each molecule gathered together, confirming the validity of the current PCA. The loading matrix obtained during the modeling process of this PCA was thus preserved for later analysis of the mixtures of the three molecules.

**Fig. 3:**
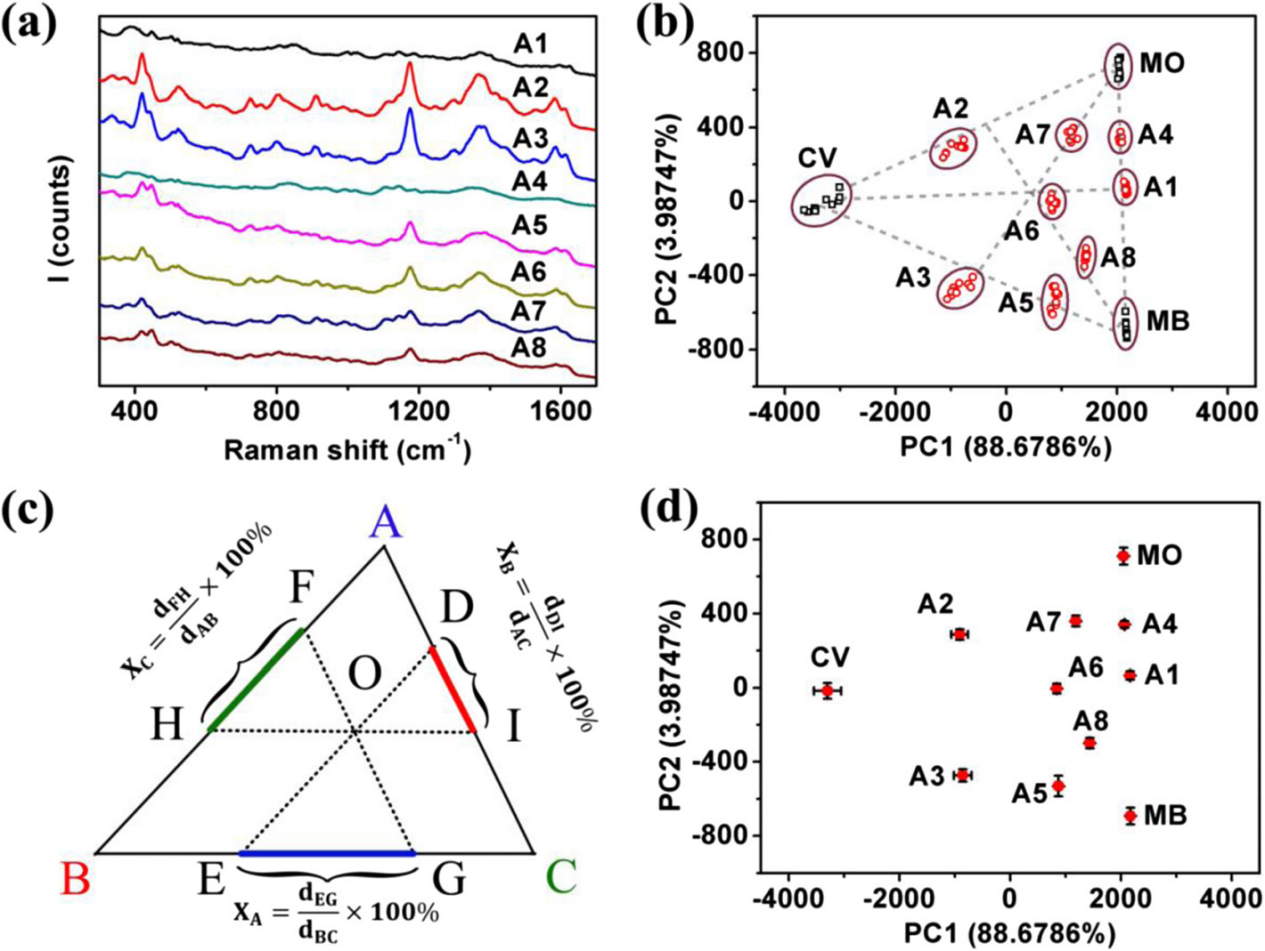
**a** SERS spectra of mixtures A1 to A8. **b** MO, MB, CV, and their mixtures’ scores. **c** Schematic plot explaining the principle of the triangle-rule. *Points A*, *B*, and *C* represent the three components, and *point O* represents their mixture. **d** Average scores of MO, MB, CV, and their mixtures

Figure 3a shows the typical SERS spectra of 5 × 10^−6^ M solutions mixed with two or three of the above molecules. Samples A1 to A5 are binary mixtures while samples A6 to A8 are ternary mixtures of MO, MB, and CV. Table 1 lists the compositions of these mixtures. Unlike the SERS spectra shown by Fig. 2a–c for pure MO, MB, and CV, the features of binary and ternary mixtures of three molecules are not so obvious. Therefore, we performed PCA for the SERS spectra of all binary and ternary mixtures using the loading matrix previously obtained during the PCA of MO, MB, and CV, and plotted the scores of PC2 versus PC1 together with those of pure MO, MB, and CV in Fig. 3b. From Fig. 3b, one notices that, by the PCA, the mixtures of MO and MB locate on the line connecting MO and MB, mixtures of MB and CV locate on the line connecting MB and CV, and mixtures of MO and CV locate on the line connecting MO and CV, respectively. For ternary mixtures of MO, MB, and CV, they locate inside the triangle formed by MO, MB, and CV. This interesting phenomenon might be understood by the following way: roughly, the SERS intensity of analyte molecules is proportional positively to their number, and PC1 and PC2 are linearly related to the SERS intensity of this analyte; thus, the PC1 and PC2 value of a binary or ternary mixture should be a weighted (the mole fraction of a specific component is the weight) sum of the corresponding values of the components. For this reason, the mole composition of a binary mixture can be calculated using the “balance-rule” with the PCA data, and the mole fraction of a ternary mixture can be calculated using the “triangle-rule” shown by Fig. 3c, where one sees clearly how the mole fraction of a specific component should be calculated. The “balance-rule” for binary mixtures can also be directly obtained from the “triangle-rule.” If it works, the qualitative PCA approach might be developed into at least a semi-quantitative analysis method for chemical mixtures by SERS. Note that the dotted lines are only for guide of the eyes.

To check out this idea, we averaged the PC1 and PC2 data of all the above samples and plotted them in Fig. 3d. Following the “triangle-rule” and “balance-rule” shown by Fig. 3c, the mole fraction of each component in all mixtures was calculated, and the data are listed in Table 2. It is noticed, surprisingly, that for the five binary mixtures (i.e., samples A1 to A5), the compositions calculated from the PCA data using the “balance-rule” are very close to their real compositions, with errors around or less than 5 %. It is also noticed that for the ternary mixtures, the calculated compositions by the “triangle-rule” are in agreement with their real compositions, with errors around or less than 5 %. The only exception is the CV composition calculated for *X*_MO_:*X*_MB_:*X*_CV_ = 1:1:1, whose error is >5 %, possibly due to the error involved during the SERS spectra acquisition. As a whole in general, the calculated compositions of all mixtures by the “balance-rule” and the “triangle-rule” are in agreement with their real compositions, confirming the validity of the “balance-rule” and “triangle-rule” proposed in this study.

**Table 2:** The mole fraction and its error of each component in all dye mixtures

Sample	MO composition (%)	Error of MO composition (%)	MB composition (%)	Error of MB composition (%)	CV composition (%)	Error of CV composition (%)
A1	53.74	3.74	46.26	3.74	…	…
A2	44.63	5.37	…	…	55.37	5.37
A3	…	…	44.98	5.02	55.02	5.02
A4	73.74	1.26	26.26	1.26	…	…
A5	…	…	76.26	1.26	23.75	1.26
A6	37.42	4.09	38.30	4.97	23.42	9.91
A7	65.96	0.70	16.19	0.48	16.47	0.20
A8	21.68	5.01	64.85	1.81	12.91	3.76

To further validate the “triangle-rule,” we calculated the compositions of ternary mixtures using the “balance-rule.” For example, as shown by Fig. 3b, d, the ternary mixture (sample A6) can also be considered as a mixture of CV and sample A1 (which is a 1:1 mixture of MO and MB). Similarly, sample A6 can also be considered as a binary mixture of MB and sample A2 (a 1:1 mixture of MO and CV) or a binary mixture of MO and sample A3 (a 1:1 mixture of MB and CV). Therefore, the composition of sample 6 (a ternary mixture) can be calculated using the “balance-rule.” Considering it as a mixture of CV and sample A1, using the “balance-rule,” the CV composition was estimated to be ~24.25 % (very close to ~23.42 % calculated by the “triangle-rule”). The compositions of MO and MB were estimated to be ~40.71 and ~35.04 %, which are calculated to be ~37.42 and ~38.30 %, respectively, by the “triangle-rule.” The agreement between the two calculations confirms the validity of the “triangle-rule” and the “balance-rule” in calculating the composition of binary and ternary mixtures from the PCA, which could be used as a semi-quantitative approach for SERS.

**Fig. 4:**
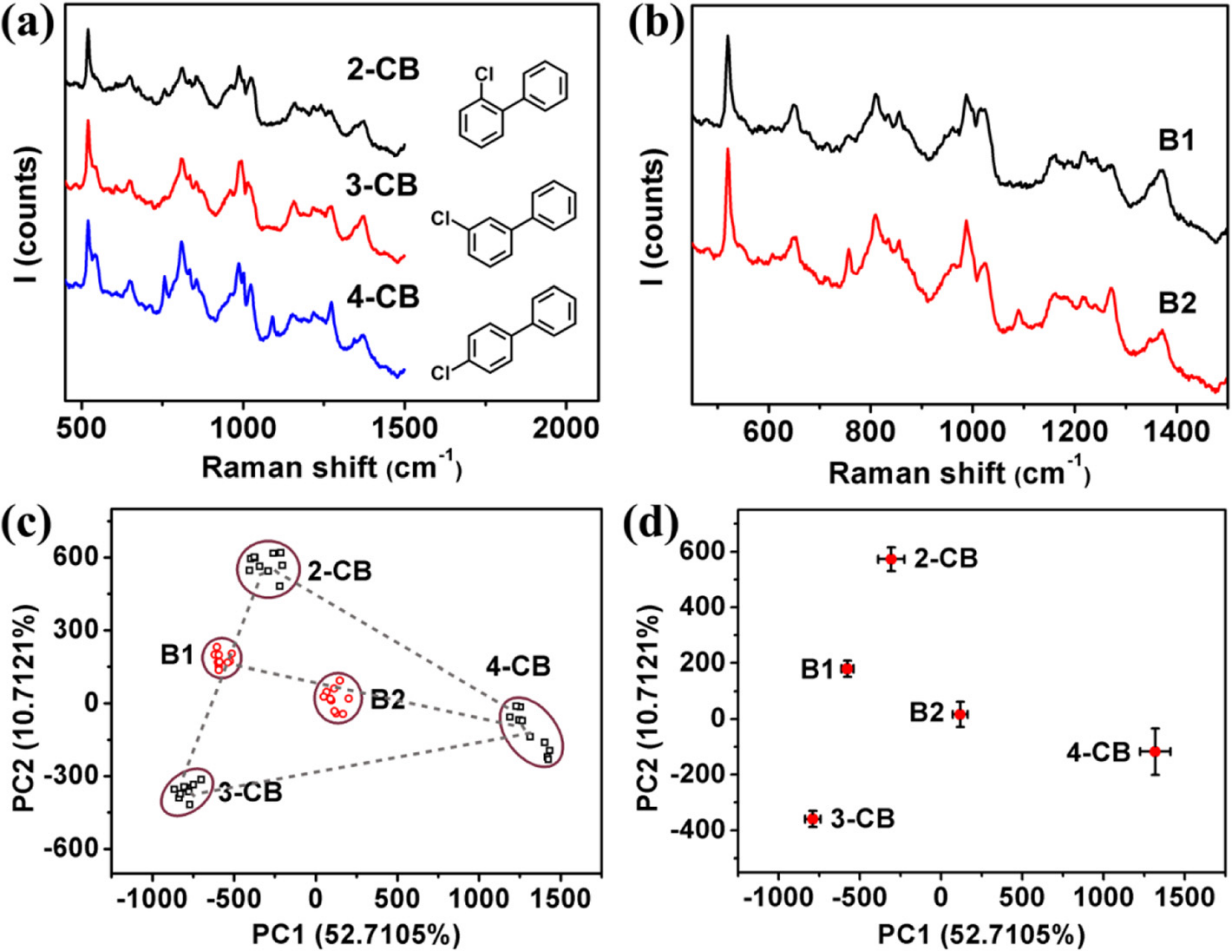
**a** Typical SERS spectrum of 5 × 10^−5^ M 2-CB, 3- CB, and 4- CB. **b** Typical SERS spectra of CB mixtures B1 and B2. **c** 2-CB, 3-CB, 4-CB, and their mixtures’ scores. **d** Average scores of 2-CB, 3-CB, 4-CB, and their mixtures corresponding to the first two principal components

PCA was also carried out for isomers of monochlorobiphenyl by SERS. It is known that monochlorobiphenyl has three isomers, i.e., 2-chlorobiphenyl (2-CB), 3-chlorobiphenyl (3-CB), and 4-chlorobiphenyl (4-CB). The only difference of the three isomers is the substitution position of chlorine atom. Figure 4a shows the typical SERS spectra of the three isomers at Raman shifts of 1274, ~1000, and ~1050 cm^−1^, corresponding to the C-C bridge bond stretching, CCC trigonal breathing, and in-plane C-H bending vibration, respectively [37, 38]. Figure 4b shows the typical SERS spectra of a binary mixture and a ternary mixture of the three isomers. It is reported that one might distinguish the three isomers directly from their Raman spectra, e.g., a Raman peak at 675 cm^−1^ for 2-CB, at 1088 cm^−1^ for 4-CB, and a strong peak at 756 cm^−1^ except for 3-CB [37, 38]. However, it is very hard to do so when their concentration is low, as shown by Fig. 4a, b. We performed PCA for the three isomers using their SERS spectra to get the loading matrix, calculated the scores for the two mixtures with the loading matrix, and plotted the obtained data together in Fig. 4c. Similarly, it is observed that the three isomers are located at different positions of the plot, and that sample B1 (a binary mixture of 2-CB and 3-CB) is located near the line connecting 2-CB and 3-CB and sample B2 (a ternary mixture of 2-CB, 3-CB, and 4-CB) is located in the triangle of 2-CB, 3-CB, and 4-CB, respectively. The data of each sample were averaged and plotted in Fig. 4d for the composition calculated using the “triangle-rule” and “balance-rule.” Table 3 lists the estimated values for the two mixtures. One sees that the calculated compositions are in good agreement with the real ones, i.e., the errors are <5 %. Again, B2 can be considered as a binary mixture of 4-CB and B1 (a mixture of 2-CB and 3-CB). Thus, we calculated the composition of sample B2 using the “balance-rule.” The estimated values are ~34.58, 28.34, and 37.08 % for 2-CB, 3-CB, and 4-CB, respectively, which are in good agreement with the values of ~31.17, 32.90, and 35.88 % by the “triangle-rule.” The above results indicate that the “triangle-rule” and “balance-rule” could be used to calculate the composition of the mixtures of chemicals of different molecule structures or similar molecule structure, by the PCA of their SERS spectra, within acceptable errors.

**Table 3:** The mole fraction and its error of each component in all monochlorobiphenyl mixtures

Sample	Concentration ratio of components	2-CB composition (%)	Error of 2-CB composition (%)	3-CB composition (%)	Error of 3-CB composition (%)	4-CB composition (%)	Error of 4-CB composition (%)
B1	*X*_2-CB_:*X*_3-CB_ = 1:1	54.96	4.96	45.04	4.96	…	…
B2	*X*_2-CB_:*X*_3-CB_:*X*_4-CB_ = 1:1:1	31.17	2.16	32.90	0.43	35.88	2.55

## Conclusions

In conclusion, we demonstrated that the PCA of the SERS spectra can be used as an effective way to distinguish the molecules in chemical mixtures qualitatively, and that it can be developed into a semi-quantitative approach to calculate the composition of binary/ternary mixtures of chemicals, using the “balance-rule” or the “triangle-rule” proposed here. Using ternary systems of MO, MB, and CV and 2-CB, 3-CB, and 4-CB as examples, we showed that the compositions of their mixtures can be calculated according to the two rules by the PCA of their SERS spectra, within acceptable errors. This study may provide a promising way to do quantitative analysis of chemical mixtures using SERS at trace levels.
